# Infective Endocarditis With Origin in Orbital Vascular Malformation and Maxillary Sinusitis: A Case Report and Review of Four Patients in the Literature

**DOI:** 10.7759/cureus.74873

**Published:** 2024-11-30

**Authors:** Toshihiko Matsuo, Yoshitaka Iwamoto, Hironori Okamoto, Daisuke Iguchi

**Affiliations:** 1 Department of Ophthalmology, Graduate School of Interdisciplinary Science and Engineering in Health Systems, Okayama University, Okayama, JPN; 2 Department of Ophthalmology, Okayama University Hospital, Okayama, JPN; 3 Department of General Internal Medicine, Okayama Medical Center, National Hospital Organization, Okayama, JPN; 4 Department of Internal Medicine, Ochiai Hospital, Maniwa, JPN

**Keywords:** infective endocarditis, maxillary sinusitis, ocular proptosis, orbital vascular malformation, streptococcus anginosus

## Abstract

Infective endocarditis is a life-threatening disease and the early diagnosis is crucial for a better outcome. We report an old adult who developed infective endocarditis in association with new-onset maxillary sinusitis as well as proptosis, which was caused by an orbital mass lesion in the background of pre-existing orbital vascular malformation. A 74-year-old woman was found incidentally to have right orbital vascular (venous) malformation by head magnetic resonance imaging when she was hospitalized for left dorsal pontine infarction. No paranasal sinusitis was noted at that time. She was well until half a year later when she developed fatigue and appetite loss for two days. At the same time, she had proptosis on the right side but did not have a fever. Blood examinations showed leukocytosis and a marked increase of C-reactive protein to 22 mg/dL as well as a moderate increase of bilirubin and liver enzymes. Emergency computed tomography scans from the head to abdomen showed nothing to be noted except for maxillary sinusitis and a retrobulbar orbital mass on the right side, which was in the same location as pre-existing vascular malformation. She began to have empirical antibiotics suspected of infective endocarditis. Head magnetic resonance imaging showed ischemic lesions in the right parietal lobe. Transthoracic and transesophageal echocardiography showed mitral valve regurgitation but no apparent vegetation. *Streptococcus anginosus* was detected by blood culture and the antibiotics were switched to intravenous penicillin G for 32 days. She was discharged in healthy condition with no proptosis. The orbital vascular malformation might serve as a route for infective endocarditis with the infectious origin in maxillary sinusitis. Maxillary sinusitis would be a predisposing factor for the development of infective endocarditis, and proptosis caused by an infectious focus of abnormal vascular channels in the orbit would lead to the early diagnosis of infective endocarditis. The present patient is unique in showing infective endocarditis in association with orbital vascular malformation.

## Introduction

Infective endocarditis is characterized by the formation of bacterial plaques called vegetation on the surface of cardiac valves, endocardium, or large vessel endothelium [1-3]. Its clinical manifestations are bacteremia or bacterial sepsis, new-onset cardiac valvular diseases, which are frequently mitral and aortic valve regurgitation, and brain infarction with emboli. Abscess in other parts of the body will develop probably by bacterial emboli. The condition is life-threatening if untreated or diagnosed in the later phase. The development of infective endocarditis associated with orbital vascular malformation has not been described up until now [4]. In this study, we present a patient who developed infective endocarditis in the background of pre-existing orbital vascular malformation and new-onset maxillary sinusitis on the same side. We also reviewed four patients in the literature who showed infective endocarditis in association with paranasal sinusitis [5-8].

## Case presentation

**Figure 1:**
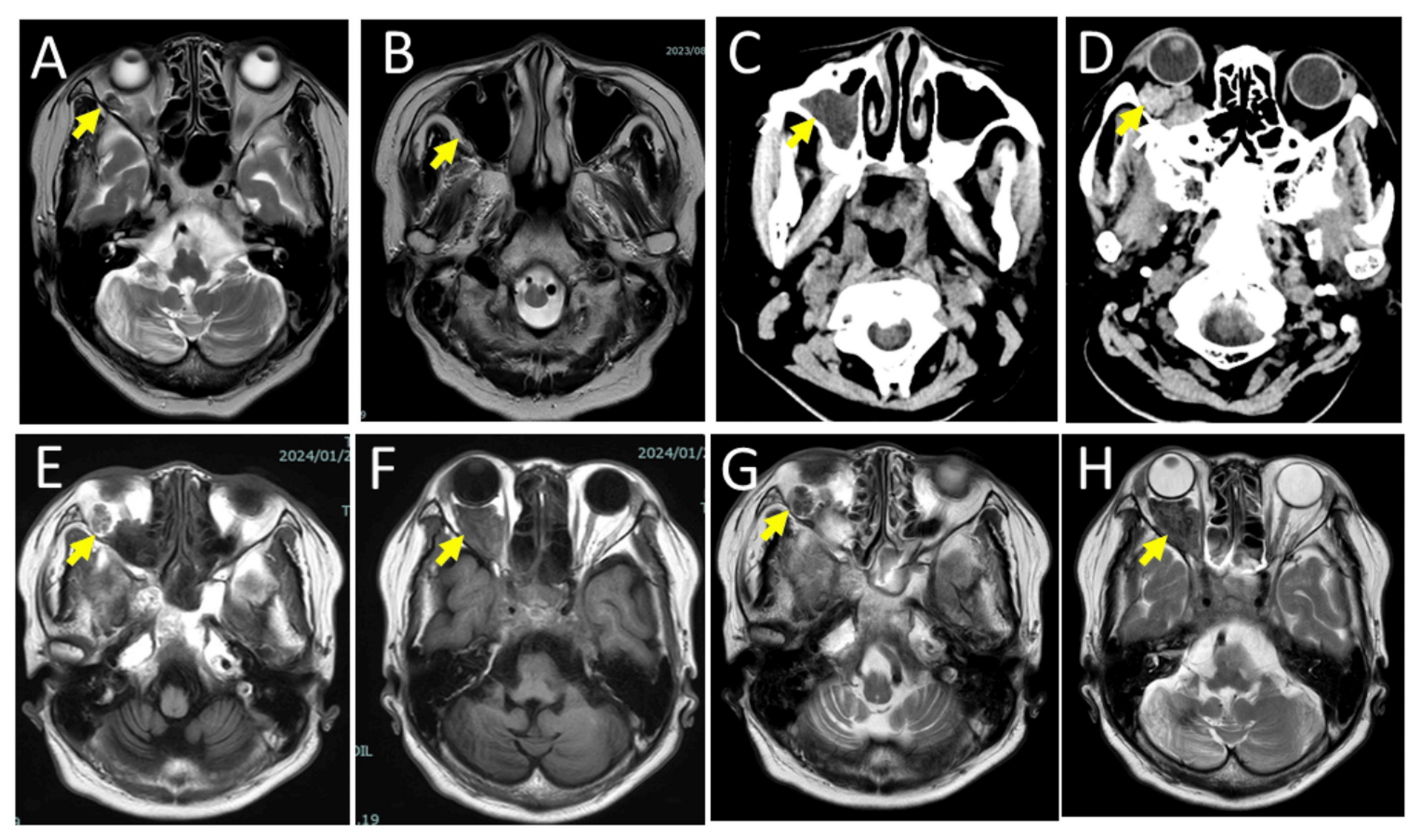
Magnetic resonance imaging half a year previously, computed tomography scans at the initial visit, and magnetic resonance imaging on the next day at referral. Incidental detection of right orbital vascular (venous) malformation (arrow, A) and the absence of right maxillary sinusitis (arrow, B) on T2-weighted magnetic resonance images half a year previously. Right maxillary sinusitis (arrow, C) and right orbital mass (arrow, D) with proptosis on emergency computed tomography scans at the initial visit to a regional hospital. Right orbital retrobulbar mass (arrows, F, H) in connection with a vascular malformation (arrows, E, G) in T1-weighted (E, F) and T2-weighted (G, H) axial images, next day at a referral hospital.

A 74-year-old woman was found incidentally to have orbital vascular (venous) malformation on the right side (Figure 1A) by head magnetic resonance imaging when she was hospitalized for left dorsal pontine infarction at a regional hospital. No paranasal sinusitis was noted at that time (Figure 1B). Since then, she recovered from mild right hemiplegia and dysarthria. She had daily oral administration of aspirin 50 mg for brain infarction, febuxostat 10 mg for hyperuricemia, pitavastatin 2 mg for dyslipidemia, lansoprazole 15 mg for gastroesophageal reflux disease, and bisoprolol fumarate 2.5 mg for hypertension. She had no other past history and did not smoke or drink alcohol.

She was well until half a year later when she developed fatigue and appetite loss for two days. At the same time, she had proptosis and upper eyelid swelling on the right side but did not have eyelid pain or redness. Systemically, she did not have a fever, nausea, abdominal pain, headache, skin rashes, or hemorrhage. Her consciousness was at a normal level. Physical examinations were normal except for mild muscle weakness of the upper and lower limbs on the right side. She maintained good oral hygiene and had no recent dental procedures. The conjunctiva in both eyes was normal without any petechiae. The best-corrected visual acuity in decimals was 1.0 in both eyes. The eye movement had no limitation, and the retina and optic discs in both eyes appeared normal. The body temperature was 36.0℃. The blood pressure was 109/66 mmHg, the pulse rate was 103/minute, and the respiration rate was 20/minute. Body weight was 46.2 kg, which was 5.3 kg lower than the measurement half a year previously. Blood examinations showed leukocytosis and a marked increase of C-reactive protein (CRP) to 22 mg/dL as well as a moderate increase of bilirubin and liver enzymes (Table 1). Urinalysis by a test tape was positive for 2+ protein, 3+ urobilinogen, and 1+ occult blood while negative for glucose. Urine sediments were positive for a small number of leukocytes while negative for bacteria by Gram stain. Suspected of the biliary tract and liver abscess, emergency computed tomography scans from the head to abdomen showed nothing to be noted except for maxillary sinusitis (Figure 1C) and an ill-defined retrobulbar orbital mass (Figure 1D) on the right side, which was on the same location of pre-existing vascular malformation (Figure 1A). Serological tests for syphilis as well as screening tests for hepatitis B virus antigen, hepatis C virus antibody, and human immunodeficiency virus antigen/antibody were all negative. Combination tests for influenza and coronavirus disease 2019 (COVID-19) antigens in nasal swabs were also negative.

**Table 1:** Blood examinations on three occasions. Electrolytes were normal on all occasions. n.d.: not determined; NT-proBNP: N-terminal pro-B-type natriuretic peptide.

	Normal range	One year previously	Onset	One month later
Red blood cells (x 10^6^/µL)	3.80-4.80	4.16	3.98	3.64
Platelets (x 10^3^/µL)	150-400	297	423	280
White blood cells (/µL)	4000-9000	7230	12,820	3700
Stab cells (%)	0.0-10.0	0	0.5	0
Segmented cells (%)	40.0-65.0	46	90	54.8
Lymphocytes (%)	25.0-45.0	47	6	31.3
Monocytes (%)	2.0-10.0	5	3.5	5.9
Eosinophils (%)	0.0-5.0	1.5	0	6.7
Basophils (%)	0.0-1.0	0.5	0	1.3
Hemoglobin (g/dL)	11.5-14.5	12.9	11.4	10.9
Hematocrit (%)	34.0-45.0	39.3	36.0	34.5
Total protein (g/dL)	6.5-8.0	8.1	6.7	5.9
Albumin (g/dL)	3.8-5.3	4.8	2.6	2.5
Lactate dehydrogenase (LD) (U/L)	124-222	226	293	134
Alkaline phosphatase (ALP) (U/L)	38-113	105	525	126
Aspartate aminotransferase (AST) (U/L)	10-35	36	65	14
Alanine aminotransferase (ALT) (U/L)	7-42	60	90	13
γ-glutamyl transferase (γ-GT) (U/L)	5-40	50	197	38
Creatine kinase (CK) (U/L)	40-180	130	365	24
Total bilirubin (mg/dL)	0.4-0.9	0.3	2.5	0.4
Urea nitrogen (mg/dL)	8.0-20.0	21.4	28.1	12
Creatinine (mg/dL)	0.45-0.80	0.81	0.86	0.68
Estimated glomerular filtration rate (eGFR) (mL/min/1.73 m^2^)	60 or greater	52	49	63
Total cholesterol (mg/dL)	130-220	241	113	n.d.
Blood glucose (mg/dL)	70-110	128	149	81
C-reactive protein (CRP) (mg/dL)	0.00-0.50	0.07	22.22	0.09
Serum Ferritin (ng/mL)	4-120	n.d.	440	n.d.
NT-proBNP (pg/mL)	0-125	n.d.	1950	447
Rheumatoid factor (IU/mL)	15 or smaller	n.d.	15	n.d.

**Figure 2:**
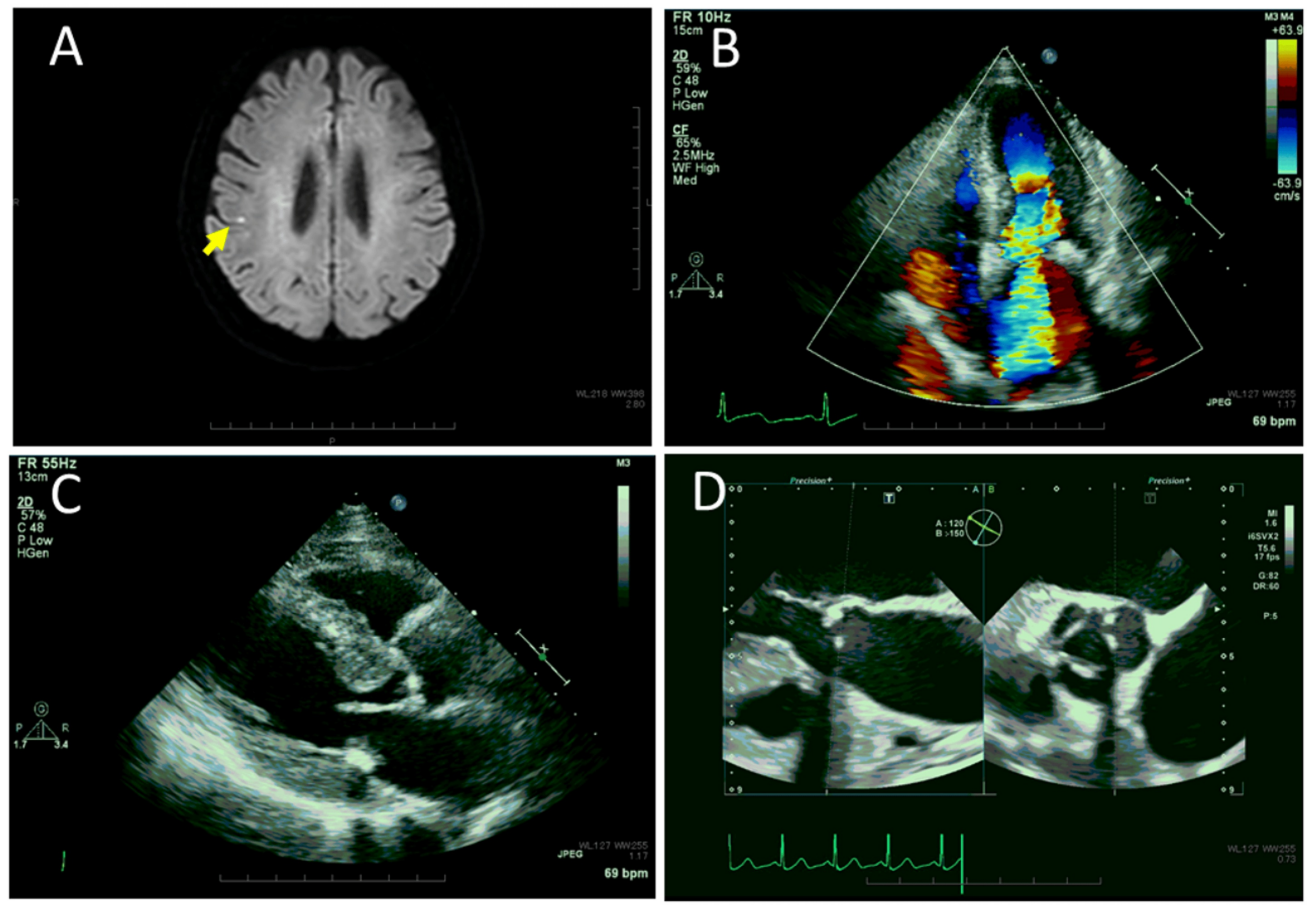
Head magnetic resonance imaging and echocardiography at referral. Head magnetic resonance imaging showing focal high-intensity spots by diffusion-weighted image in the right parietal lobe (arrow, A), indicative of ischemic lesions. Doppler imaging of transthoracic echocardiography (B) showing mitral valve regurgitation. Transthoracic echocardiography (C) showed no apparent vegetation on the long-axis view of the aortic valve and mitral valve. Transesophageal echocardiography (D) showed no apparent vegetation on the long-axis view (left panel) and short-axis view (right panel) of the aortic valve.

**Figure 3:**
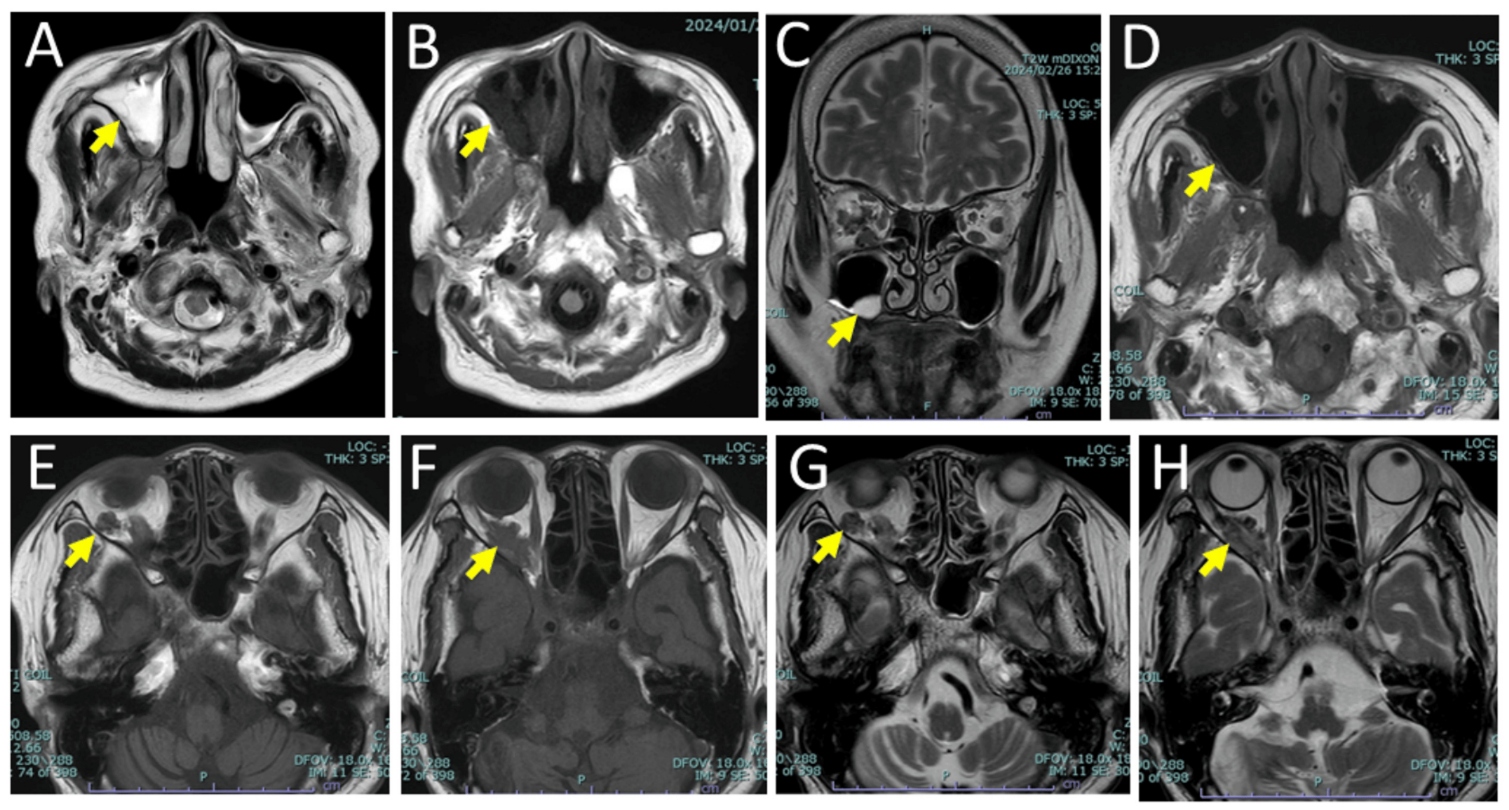
Magnetic resonance imaging on the next day at referral and one month later. Right maxillary sinusitis (arrows) in T2-weighted (A) and T1-weighted (B) axial images the next day at a referral hospital. Resolution of right maxillary sinusitis (arrows) in T2-weighted coronal (C) and T1-weighted axial (D) images in one month with penicillin G treatment. Note a small volume of residual fluid in the right maxillary sinus (arrow, C). Right orbital vascular malformation (arrows) in the pre-existing size and no proptosis after one month of antibacterial treatment in T1-weighted (E, F) and T2-weighted (G, H) axial images.

She was hospitalized later on the same day at a referral hospital for further examination and treatment. At referral, the body temperature was elevated to 38.9℃, the blood pressure was 128/71 mmHg, the heart rate was regular at 90/minute, and the respiratory rate was 22/minute. No Osler nodule was found by physical examination and a systolic murmur was heard on the 3/6 Levine grading scale by auscultation. As an empiric therapy, she began to have intravenous piperacillin/tazobactam, suspected of infective cholangitis. Magnetic resonance cholangiopancreatography, however, disclosed no infectious focus. On the next day, the treatment was switched to intravenous ampicillin and vancomycin, suspected this time of infective endocarditis because Gram-positive chain-forming cocci were detected by a smear of blood culture. Head magnetic resonance imaging revealed focal high-intensity spots by diffusion-weighted images in the right parietal lobe (Figure 2A), indicative of ischemic lesions, and also showed a right orbital retrobulbar mass (Figures 1F, 1H) with a tubular structure (Figures 1E, 1G) and right maxillary sinusitis (Figures 3A, 3B). Transthoracic and transesophageal echocardiography showed mitral valve regurgitation (Figure 2B) but revealed no apparent vegetation (Figures 2C, 2D). On the sixth day of hospitalization, *Streptococcus anginosus* was identified by VITEK 2 COMPACT automated microbial detection system (bioMerieux, Marcy-l'Étoile, France) in growth from two bottles each for aerobic and anaerobic conditions of blood culture and showed the minimum inhibitory concentration of penicillin G ≤ 0.06. The antibiotics were, thus, switched to continuous intravenous penicillin G at 24 million units daily for 32 days to ensure the clearance of bacteremia. The right maxillary sinusitis cleared (Figures 3C, 3D) and the right orbital retrobulbar mass lesion resolved with the pre-existing vascular malformation left behind (Figures 3E-3H). She was discharged with no proptosis in healthy condition (Table 1) and had daily administration of amoxicillin 1500 mg for four weeks to completely eradicate the bacterial focus for bacteremia. The mitral valve regurgitation was followed with no treatment since she did not have symptoms. She returned to routine follow-up every half a year at the regional hospital.

## Discussion

Based on the modified Duke criteria for the diagnosis of infective endocarditis [2,3], the present patient showed positive blood culture in two separate bottles as the major criteria. Intracardiac vegetation was not detected by transthoracic and transesophageal echocardiography. The mitral valve regurgitation might be a newly developed sign or might have been present in the circumstances that a systolic murmur was heard on auscultation at the referral. A limitation is that transthoracic echocardiography had not been done in this patient before the onset of the present symptoms. As for the minor criteria, she showed ischemic brain lesions, which might be caused by embolization. Qualitative urinalysis at the onset revealed urinary occult blood and protein, suspicious of glomerulonephritis. She presented a high fever at the referral and might have a predisposition to mitral valve regurgitation by echocardiography. Several hours previously at the initial visit to the regional hospital, she had no fever, even though she showed leukocytosis and a marked increase of CRP as severe inflammatory signs. The delayed onset of fever would be attributed to her old age. From the ophthalmic point of view, she did not show conjunctival petechiae or retinal hemorrhages, including Roth spots, suggestive of infective endocarditis. Accordingly, the present patient fulfilled one major criterion (positive blood culture) and three minor criteria (mitral valve predisposition, fever, and brain embolism), leading to the diagnosis of definite infective endocarditis. Probable glomerulonephritis, based just on urinalysis, would be also considered as a minor criterion. When the mitral valve predisposition is excluded, the diagnosis would be possible infective endocarditis.

The present patient is unique in the point that she developed infective endocarditis in association with new-onset maxillary sinusitis and proptosis caused by orbital mass formation on the same right side. The absence of maxillary sinusitis and the presence of orbital vascular malformation had been confirmed by magnetic resonance imaging half a year before the onset of infective endocarditis. Furthermore, both maxillary sinusitis and orbital mass formation resolved in a month with antibacterial treatment for infective endocarditis, leaving behind the pre-existing orbital vascular malformation. The infective endocarditis, maxillary sinusitis, and orbital mass formation in association with pre-existing vascular malformation might be related to one another or might otherwise be combined simply by chance in this patient.

To analyze similar cases, PubMed and Google Scholar were searched with keywords including “endocarditis” and “paranasal sinusitis”. A sufficient description was found in four patients (Table 2) [5-8]. All four patients with infective endocarditis as a sequel to paranasal sinusitis were men with the onset age in the 20s and 30s in three patients and 70s in the remaining one (case 3), as in the present patient (case 5 in Table 2). Three younger patients developed fever and stated no intravenous drug abuse while one older patient (case 3) had no fever, as in the present patient (case 5). As for the pathogen, *Haemophilus parainfluenzae* was detected by blood culture in two patients and *Streptococcus pneumoniae* in the other two patients. All three young patients had mitral valve involvement with apparent vegetation and concurrently showed multiple ischemic brain lesions. In addition, one (case 4) of these three young patients was diagnosed with bacterial meningitis. These three young patients finally underwent mitral valve repair surgery. In contrast, one older patient (case 3) showed mild aortic valve regurgitation with vegetation and lumbar paravertebral abscess but did not require surgical intervention. The present patient (case 5 in Table 2) also did not have surgical intervention.

**Table 2:** Review of five patients with infective endocarditis with its origin in paranasal sinusitis, including the present patient. No patient reported intravenous drug abuse.

Case No./gender/age at onset	Presenting symptoms	Blood culture and other culture	Transthoracic and transesophageal echocardiography	Brain MRI and other MRI	Paranasal sinusitis on CT or MRI	Treatment	Other features and outcome	Author (year)
1/male/36	Two-week-long seromucous nasal discharge, productive cough, fatigue, myalgia, fever up to 39℃	Haemophilus parainfluenzae	Mild thickening of mitral valve, mitral regurgitation by prolapse and vegetation a week later	Multiple ischemic lesions	Left maxillary sinusitis	Empirical amoxicillin clavulanate, intravenous ceftriaxone 2 g daily for 6 weeks	Cocaine abuse, mitral valve repair surgery	Barreto Cortes et al. (2016) [5]
2/male/27	One-week-long severe headache, intermittent fever, chills, fatigue	*Haemophilus parainfluenzae,* cerebrospinal fluid negative for bacteria	Mitral valve regurgitation with perforation and vegetation	Multiple ischemic lesions	Right maxillary sinusitis	Empirical vancomycin and aztreonam, intravenous ceftriaxone 2 g daily for 6 weeks	Mitral valve repair surgery in plan	Duzenli et al. (2017) [6]
3/male/71	One-week-long back pain and right ankle pain, right ankle erythema	Streptococcus pneumoniae	Mild aortic valve regurgitation, vegetation-like lesion in the right coronary cusp	Lumbar paravertebral abscess	Bilateral maxillary sinusitis	Empirical gentamycin and vancomycin, intravenous ceftriaxone 2 g daily, switched to ampicillin due to skin rashes	None	Yamazaki et al. (2022) [7]
4/male/20s	Fever, headache, fatigue, nasal congestion, appetite loss, nausea, vomiting, diarrhea	*Streptococcus pneumoniae,* cerebrospinal fluid positive for *Streptococcus* *pneumoniae*	Mitral valve regurgitation, mobile valvular vegetation	Multiple ischemic lesions, bacterial meningitis	Left maxillary sinusitis	Empirical vancomycin and dexamethasone, intravenous ceftriaxone for 8 weeks	Cocaine abuse, mechanical mitral valve replacement	Erdem et al. (2023) [8]
5/female/75	Fatigue, appetite loss, right proptosis, no fever	Streptococcus anginosus	Mitral valve regurgitation, vegetation not clearly detected	Ischemic lesions in the right parietal lobe	Right maxillary sinusitis	Empirical ampicillin and vancomycin switched to intravenous penicillin G for 32 days, and finally to oral ampicillin for 4 weeks	Pre-existing right orbital vascular (venous) malformation	This case

All four patients in the literature showed infective endocarditis in association with maxillary sinusitis [5-8]. At the onset of infective endocarditis, the present patient also showed maxillary sinusitis, which had been absent half a year previously. She developed proptosis caused by enlargement of the pre-existing orbital vascular malformation in the early phase of infective endocarditis. Infective endocarditis, as a result of maxillary sinusitis, would lead to bacterial infection of abnormal vascular channels in the orbit, which would have a slow blood flow. This sequence of events is supported by the case report that infective endocarditis would develop orbital cellulitis [9]. In the present patient, however, the absence of pain and redness in the eyelid was different from typical manifestations of orbital cellulitis.

It has been known that the orbit and neighboring paranasal sinuses have vascular anastomosis [10]. The orbital vascular malformation might have an anastomotic connection with vessels in the maxillary sinus. In this situation, bacterial infection of maxillary sinusitis would at first lead to the infection of orbital vascular malformation and then would proceed to systemic bloodstream dissemination as infective endocarditis. Indeed, the probable connection of the paranasal sinus with the orbit would be supported by a case report describing that orbital myxoma had a connection with paranasal sinuses [11]. The absence of eyelid pain and redness in the present patient suggested the closed-space infection in abnormal vascular channels of the orbit, rather than diffuse orbital infection as orbital cellulitis.

*Streptococcus anginosus*, which was detected as a pathogen in the present patient, is a part of the resident flora in the oral and nasal cavity. There have been case reports [12,13] as well as a large cohort study [14] and a review [15] that described *Streptococcus anginosus* as a causative agent for infective endocarditis. The present patient did not have other predisposing factors as a risk for infective endocarditis such as periodontal diseases [1-3,16]. The orbital vascular malformation with no symptoms and signs that had been found incidentally half a year before the onset of infective endocarditis appeared to be venous [4], based on a tubular feature by magnetic resonance imaging. The venous malformation with low blood flow would tend to become a focus for infection [17,18]. In the present patient, the pre-existing orbital venous malformation would serve as a local focus for bloodstream infection in the early phase and would lead to the early development of a sign of proptosis. Under the circumstances, we could reach an early diagnosis of infective endocarditis in this patient.

## Conclusions

The present patient developed infective endocarditis with *Streptococcus anginosus* that was associated with new-onset maxillary sinusitis and proptosis caused by an orbital mass lesion in the background of pre-existing vascular malformation. Presumably, bacterial infection of maxillary sinusitis would at first lead to the infection of orbital vascular malformation and then would proceed to systemic bloodstream dissemination as infective endocarditis. In the literature, four patients with infective endocarditis were reported in association with maxillary sinusitis, the same as the present patient. Maxillary sinusitis would be a predisposing factor for the development of infective endocarditis, and proptosis in the present patient caused by an infectious focus of abnormal vascular channels in the orbit would lead to the early diagnosis of infective endocarditis. To the best of our knowledge, this patient is the first to develop infective endocarditis in association with orbital vascular malformation.
